# A Novel Augmented Reality Navigation System for Endoscopic Sinus and Skull Base Surgery: A Feasibility Study

**DOI:** 10.1371/journal.pone.0146996

**Published:** 2016-01-12

**Authors:** Liang Li, Jian Yang, Yakui Chu, Wenbo Wu, Jin Xue, Ping Liang, Lei Chen

**Affiliations:** 1 Department of Otolaryngology-Head and Neck Surgery, Chinese PLA General Hospital, Beijing, China; 2 Beijing Engineering Research Center of Mixed Reality and Advanced Display, School of Optics and Electronics, Beijing Institute of Technology, Beijing, China; 3 Department of Interventional Ultrasound, Chinese PLA General Hospital, Beijing, China; Medical University of South Carolina, UNITED STATES

## Abstract

**Objective:**

To verify the reliability and clinical feasibility of a self-developed navigation system based on an augmented reality technique for endoscopic sinus and skull base surgery.

**Materials and Methods:**

In this study we performed a head phantom and cadaver experiment to determine the display effect and accuracy of our navigational system. We compared cadaver head-based simulated operations, the target registration error, operation time, and National Aeronautics and Space Administration Task Load Index scores of our navigation system to conventional navigation systems.

**Results:**

The navigation system developed in this study has a novel display mode capable of fusing endoscopic images to three-dimensional (3-D) virtual images. In the cadaver head experiment, the target registration error was 1.28 ± 0.45 mm, which met the accepted standards of a navigation system used for nasal endoscopic surgery. Compared with conventional navigation systems, the new system was more effective in terms of operation time and the mental workload of surgeons, which is especially important for less experienced surgeons.

**Conclusion:**

The self-developed augmented reality navigation system for endoscopic sinus and skull base surgery appears to have advantages that outweigh those of conventional navigation systems. We conclude that this navigational system will provide rhinologists with more intuitive and more detailed imaging information, thus reducing the judgment time and mental workload of surgeons when performing complex sinus and skull base surgeries. Ultimately, this new navigational system has potential to increase the quality of surgeries. In addition, the augmented reality navigational system could be of interest to junior doctors being trained in endoscopic techniques because it could speed up their learning. However, it should be noted that the navigation system serves as an adjunct to a surgeon’s skills and knowledge, not as a substitute.

## Introduction

**Fig 1:**
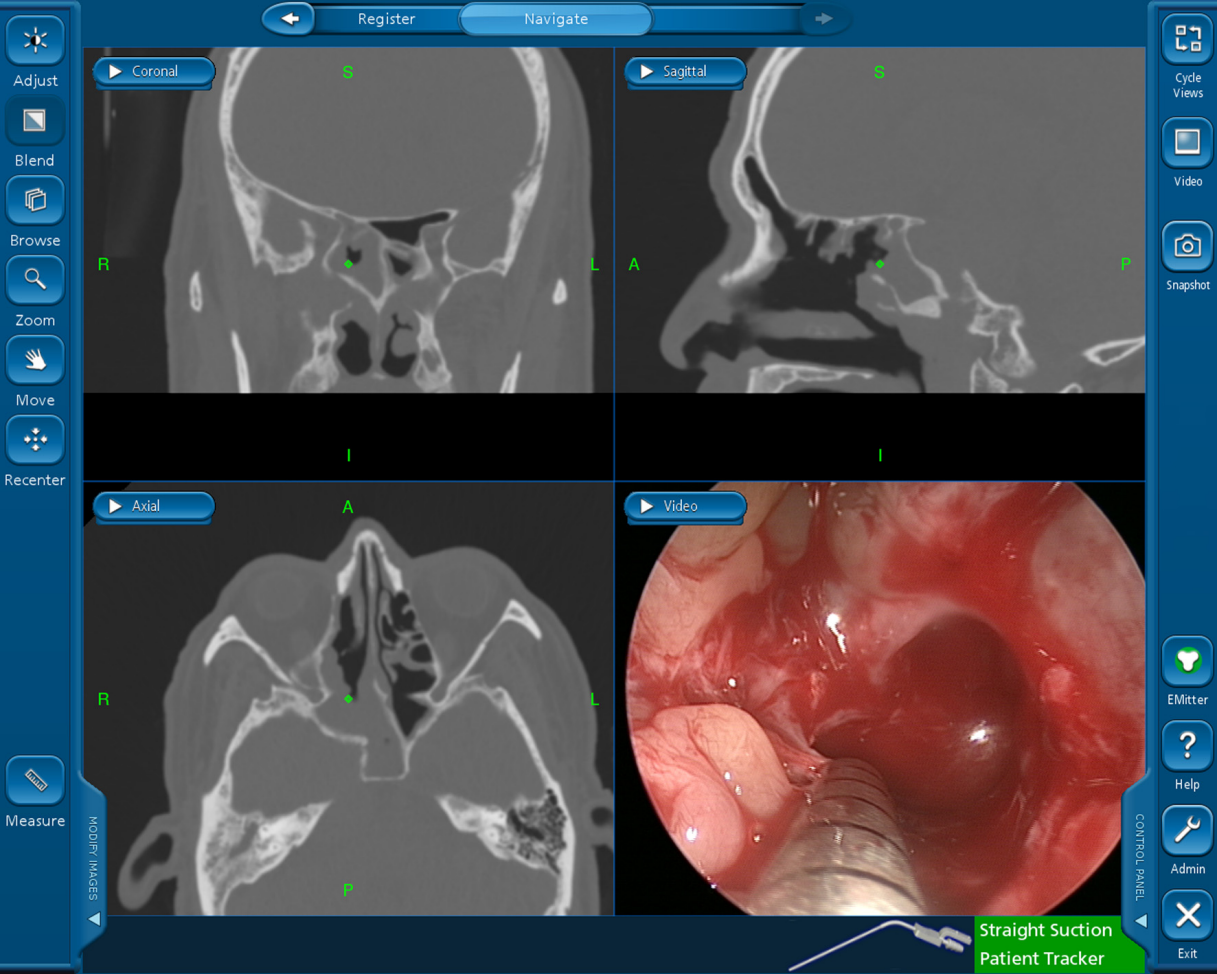
Triplanar view mode of the conventional navigation system.

**Fig 2:**
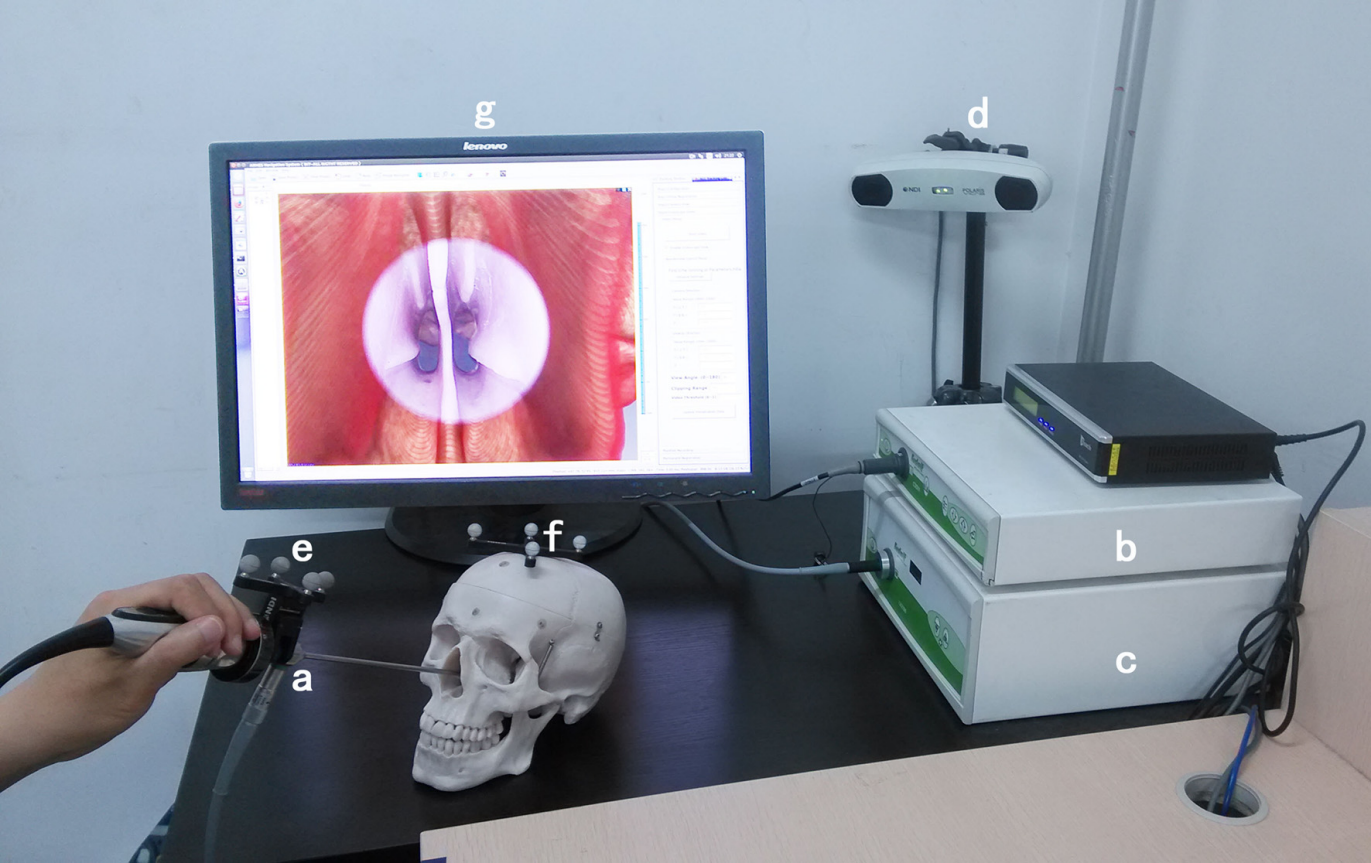
Components of the augmented reality navigation system. Nasal endoscopy imaging system: a. 0° endoscope, b. video converter, c. light source. Tracking system: d. infrared emission device, e and f. reflection markers fixed on the endoscope and the patient’s head. Workstation: g. the liquid-crystal display.

Endoscopic sinus surgery techniques have expanded and are now used to treat orbital and skull base diseases. However, an increasing number of complications related to nasal endoscopic surgery have been reported, including severe bleeding, blindness, and damage to the central nervous system. To address these concerns, navigational systems for nasal endoscopic surgery have been developed and used in clinics to prevent and reduce endoscopy-related issues [1]. However, correlative research showed that the benefits conferred by navigation systems are extremely limited [2]. Based on our review of the literature and personal experience, we determined that navigational systems fail for the following reasons: (1) it is difficult for doctors to match the tomographic image on the screen to the actual structures in the operated area, especially for doctors with less experience (Fig 1); (2) in conventional navigation (C-N) systems, images are displayed on another screen, so the surgeon has to look away from the surgical field when the anatomical location is being defined, which can be dangerous during key surgical steps [3]; and (3) a great amount of time is spent repeatedly verifying surgical sites using a probe during the operation.

Due to these limitations, we developed an augmented reality (AR)-based navigation system for endoscopic sinus and skull base surgery. In this system, three-dimensional (3-D) virtual images, based on preoperative computed tomography scans or/and magnetic resonance imaging (CT/MRI) images, are used as the background, and the distortion-corrected real endoscopic images are semi-transparently fused to it. This new display mode allows surgeons to stereoscopically observe the subsurface and surrounding anatomical structures of the surgical field, providing more detailed and intuitive information for safer surgeries. In this study, we used a series of experiments to evaluate the accuracy of our AR navigation (AR-N) system and to demonstrate its advantages, analyze its benefits to surgeons, and determine its clinical feasibility.

## Materials and Methods

### 1. Platform setup

**Fig 3:**
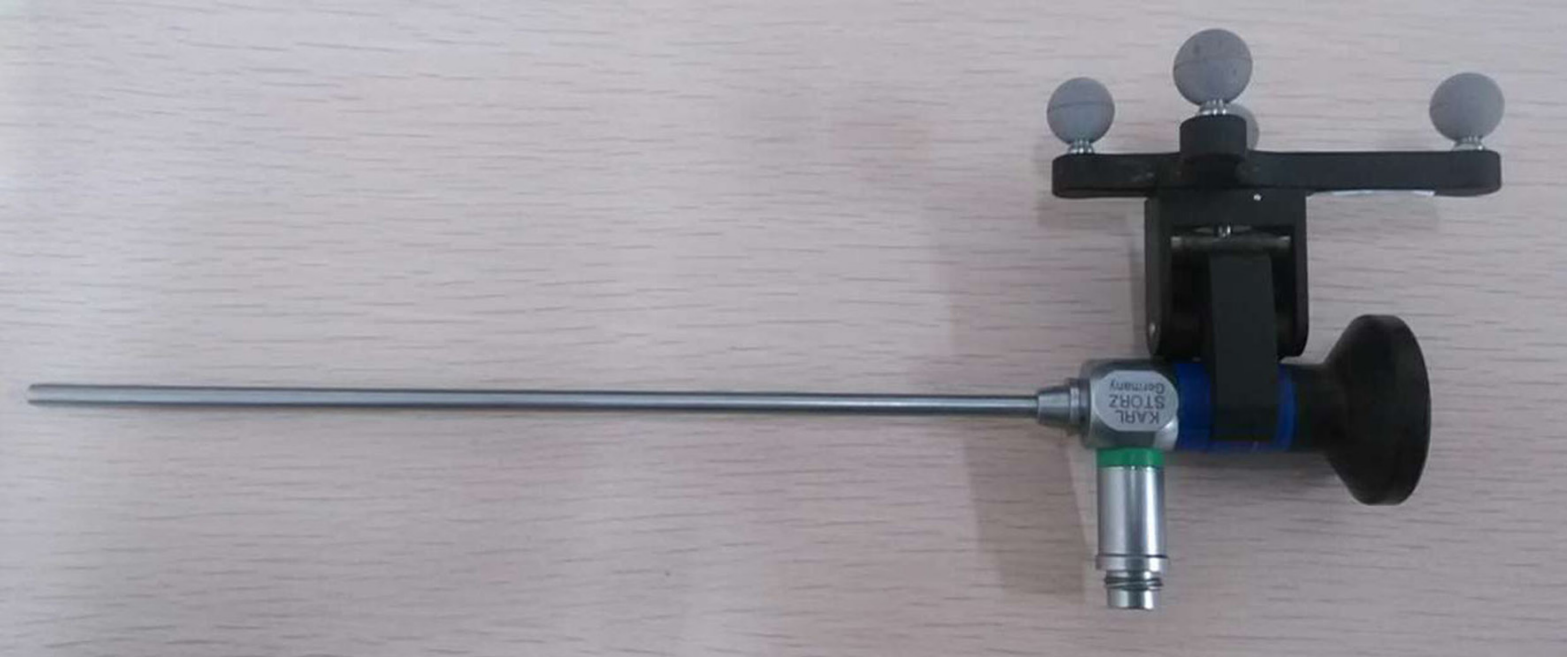
Reflection marker fixed to the endoscope. The marker allows for real-time lens tracking during surgery.

The AR-N system consists of the following components (Fig 2): (1) nasal endoscopy imaging system (RU0101-25, Rudolf Medizintechnik GmbH & Co. KG, Tuttlingen, Germany) and 0° endoscope (Karl Storz, Tuttlingen, Germany); (2) tracking system POLARIS Vicra (Northern Digital Inc., Waterloo, Canada) including an infrared emission device and reflection markers fixed on the endoscope (Fig 3) and the patient’s head; and (3) a workstation (Intel Core i7) equipped with a self-developed open-source software.

The newly developed navigation system has a similar configuration to conventional systems. However, improvements include binding of the reflection markers to the endoscope, a novel navigation software package that can track the movement of the endoscope, and a merging of the operative view with stereoscopic 3-D virtual images of anatomical structures.

During system design, we ensured that real endoscopic images could not be seamlessly superimposed onto the virtual model until radial distortion of endoscopic images was corrected. We utilized a spherical surface stereographic projection technique, as described in our previous study [4]. After estimating the field of view of the endoscope by detecting the corners, the coefficients of the spherical projection model are fitted using the least-squares method and the distorted images can be corrected according to the spherical projection maps. The method is fully automatic and requires no human intervention.

### 2. Experimental procedures

**Fig 4:**
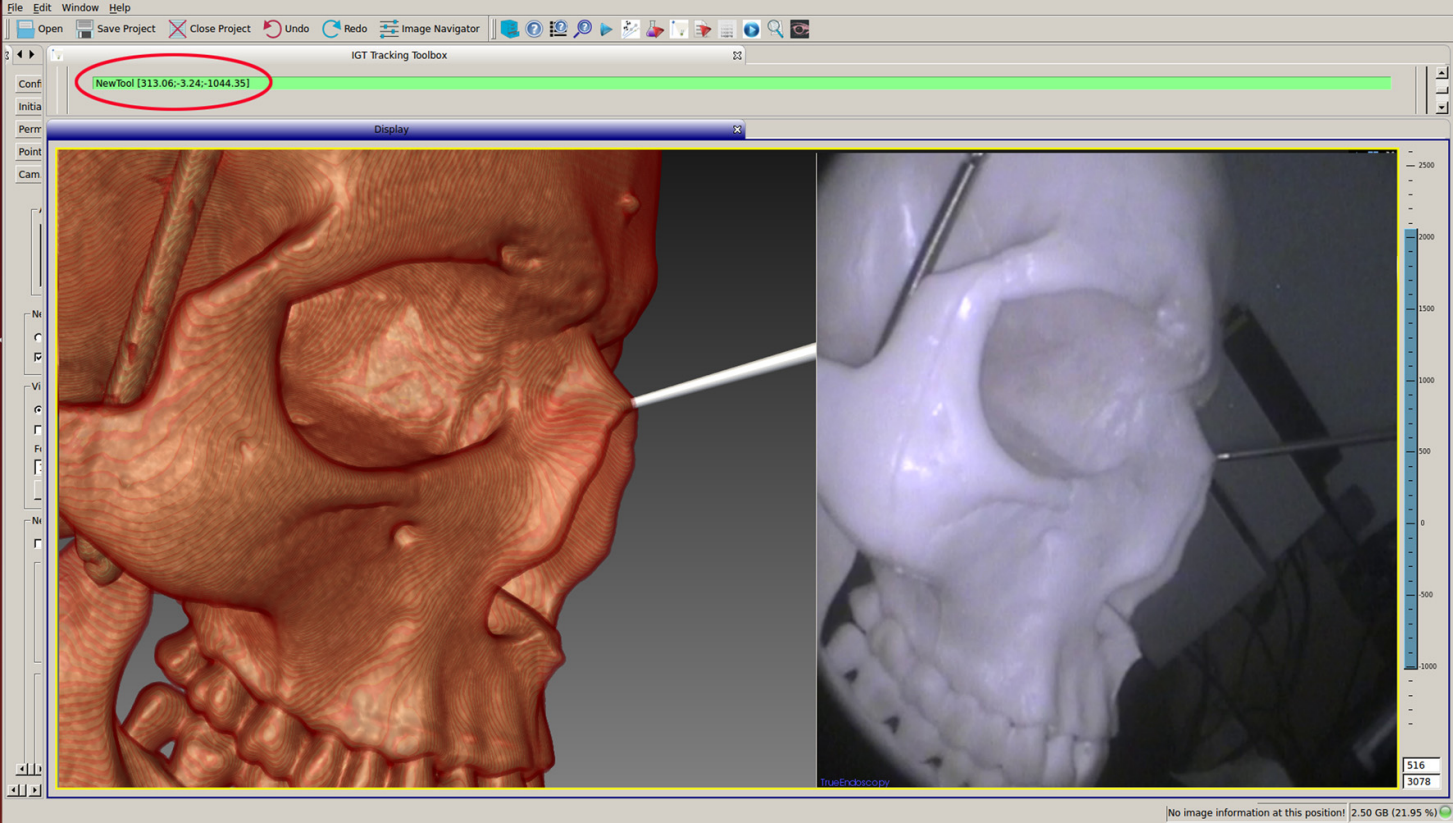
3-D coordinates obtained by pointing to the piriform aperture vertex with the probe tip.

To evaluate the accuracy of our system, we used a PVC qualitative skull phantom with an openable top with six non-coplanar reference markers, which was previously determined as the most appropriate number of markers [5]. These markers were fixed at the parietal, frontal, and bilateral temples and mastoid processes. A CT scan (thickness 0.75 mm) (GE Brightspeed Elite, General Electric Company) was performed to obtain imaging data. The data were transferred into the workstation and the skull phantom with reflection markers was fixed to the operating platform. Reference markers were individually touched with an accurately calibrated endoscope tip by the participants; corresponding points on the virtual image were then selected to complete registration. Participants were then asked to use the calibrated probe, which was used only for the test, to point to the center of specific anatomical markers (e.g., piriform aperture vertex, bilateral supraorbital foramen, the anterior end of the inferior turbinate attachment, anterior clinoid process and posterior clinoid process: nine points in total) to obtain the 3-D coordinates of those points (Fig 4). The probe was then moved slightly so that its virtual tip on the screen was pointing at the corresponding position on the virtual image to obtain another 3-D coordinate. The spatial distance between these two coordinates was calculated as the target registration error (TRE). After measuring the nine points, the average TRE was calculated and recorded, with this whole process representing a single test. If the distance obtained was smaller than 2 mm, the recognized allowable error in navigation systems for nasal endoscopic surgeries [6], the points were considered as accurately corresponding between the real and virtual images (i.e., the two images can be perfectly superimposed). A total of nine participants took the test, all of whom were asked to complete the test on nine separate occasions; therefore, we obtained 81 average TRE values. Each operation included rebooting the workstation, restarting the programs, and registering the phantom. Experimental results were recorded and analyzed.

The above results are theoretical values based on a rigid model. To evaluate the system’s performance in practice, we utilized 15 fixed cadaver heads to simulate surgery aided by the system. We compared several key indexes about the procedure from our navigational system with those of a C-N system. The experiment was performed after receiving ethical approval from the Committee on Ethics of Biomedicine Research at Chinese PLA General Hospital (Beijing, China). Experimental cadaver heads were provided by the Beijing Society for Anatomical Sciences (http://test.bjmu.edu.cn/). The process of donating remains to the society involves the donating individuals and their families submitting an application and providing written informed consent to participate in medical research. The Ethics Committee confirmed that the cadaver heads were being used legally and that the entire research process satisfied all ethical requirements. Prior to the experiment, the arteries were infused with a mixture of paint, latex, and developing agent to plump them and allow observation of the surgical field and images. Image collection, data processing, and registration before and prior to the operation were performed as for the phantom experiment. A total of 15 otorhinolaryngologists participated in this study, and each performed maxillary sinus expansion, ethmoidectomy, frontal sinus expansion, sphenoidotomy (Messerklinger method was used as the standard), and intracavernous internal carotid artery anatomical dissection on both nasal cavities of one cadaver head, guided on one side by AR-N and on the other by C-N. The order of sides and order of navigation system employment for each specimen was randomly assigned. TRE and operation time (OT) were recorded after each operation. Meanwhile, to determine the surgeon’s perceived workload and mental demands, a dialog-based computer application developed by Cao et al. [7] based on the National Aeronautics and Space Administration Task Load Index (NASA-TLX) (Table 1) was used during performance of the operation on each side. After the operation, each surgeon completed a computer-based survey to rate his or her experience. At the end of the experiment, the quantified scores in each subscale and the overall score obtained through the program were used to assess the level of assistance provided by the navigational systems. Data were statistically analyzed using IBM SPSS Statistics version 22, and *P* < 0.05 was considered statistically significant.

**Table 1:** Description of the NASA-TLX rating scale [7].

Title	Description
Mental Demand	How much mental and perceptual activity was required (e.g., thinking, deciding, calculating, remembering, looking, searching)? Was the task easy or demanding, simple or complex, exacting or forgiving?
Physical Demand	How much physical activity was required (e.g., pushing, pulling, turning, controlling, activating)? Was the task easy or demanding, slow or brisk, slack or strenuous, restful or laborious?
Temporal Demand	How much time pressure did you feel due to the rate or pace at which the tasks or task elements occurred? Was the pace slow and leisurely or rapid and frantic?
Performance	How successful do you think you were in accomplishing the goals of the task set by the experimenter (or yourself)? How satisfied were you with your performance in accomplishing these goals?
Effort	How hard did you have to work (mentally and physically) to accomplish your level of performance?
Frustration Level	How insecure, discouraged, irritated, stressed, and annoyed or secure, gratified, content, relaxed, and complacent did you feel during the task?

## Results

**Fig 5:**
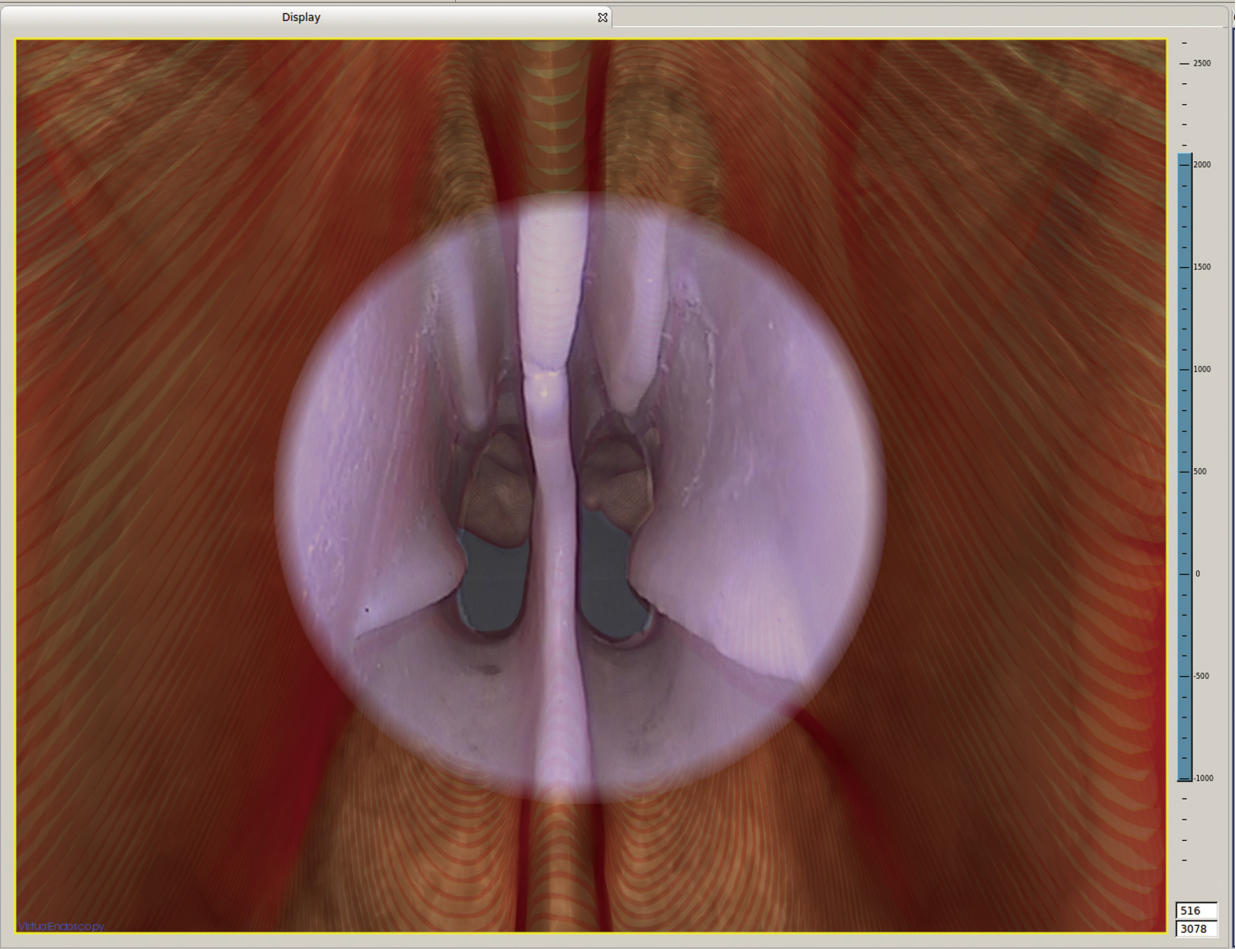
Display fusing the real nasal endoscopic images with 3-D virtual images in the head phantom experiment. The center of the display shows the endoscopic image, and the surrounding background is the 3-D virtual image.

**Fig 6:**
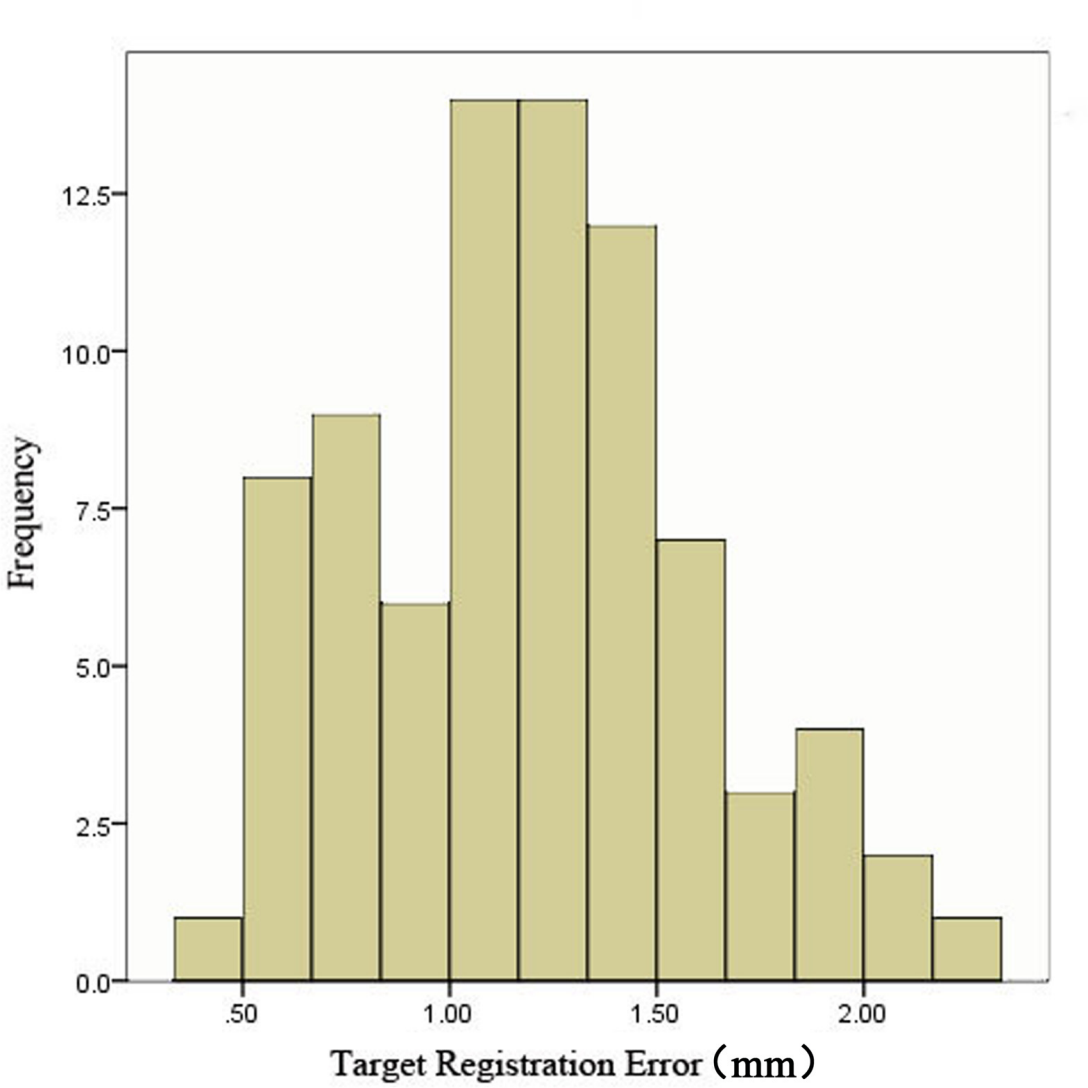
Frequency distribution of target registration error (TRE) during the head phantom experiment.

The newly developed AR-N system display mode was able to fuse nasal endoscopic images into 3-D virtual images. The head phantom experiment demonstrated the satisfactory display effect (Fig 5): when we examined the nasal cavity with the 0° lens, as mentioned in the Materials and Methods section, the contours of the anatomical structure on both images overlapped accurately (S1 Video). Moreover, by measuring nine different points for each of the nine participants (S1 Table), the average TRE of the system was calculated as 1.19 ± 0.42 mm. The frequency distributions of all of the recorded data are shown in Fig 6.

**Fig 7:**
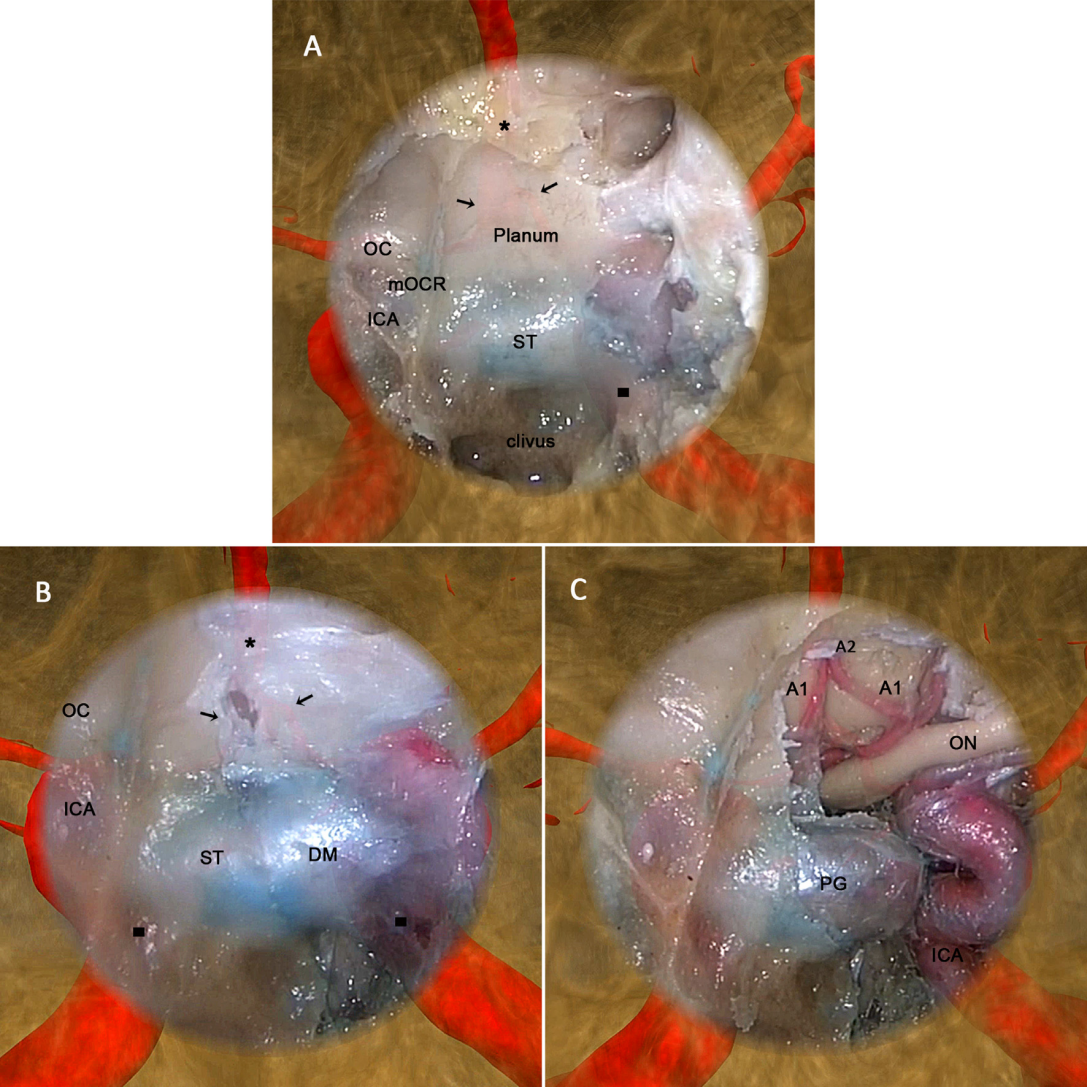
The augmented reality navigation system display during the cadaver head experiment. After maxillary sinus expansion, ethmoidectomy, and frontal sinus expansion, a wide sphenoidotomy using an endoscopic endonasal approach was performed as the final part of the operation. Moreover, to provide sufficient exposure and maneuverability, the posterior nasal septum and middle turbinate were removed so that the classic intrasphenoid landmarks could be identified (A) involving the planum sphenoidale: OC, optic canal; mOCR, medial optico-carotid recesses; ICA, internal carotid artery; ST, sella turcica; and clivus. In addition, with image superimposition, we could observe the projection of certain anatomic structures on and around the endoscopic image, involving the A1 (black arrows) and A2 (black stars) segments of the anterior cerebral artery and internal carotid artery (black squares). In fact, these structures included any of concern to the surgeon, as long as they were segmented from the computed tomography (CT) image manually or automatically before the operation. (B) Bone in the left superior, posterior, and lateral walls of the sphenoid sinus was removed to expose the dura mater. As the lens moved forward, the projection of the bilateral internal carotid artery became much clearer. (C) After the dura mater was opened, the A1 and A2 segments of the anterior cerebral artery and left internal carotid artery were exposed; the actual locations were consistent with their projections, showing that the virtual and real images were fused accurately and moved synchronously. The AR-N system display expands the surgical field during nasal endoscopy both in terms of depth and breadth, and can thus provide surgeons with more intuitive information regarding anatomical structures.

**Fig 8:**
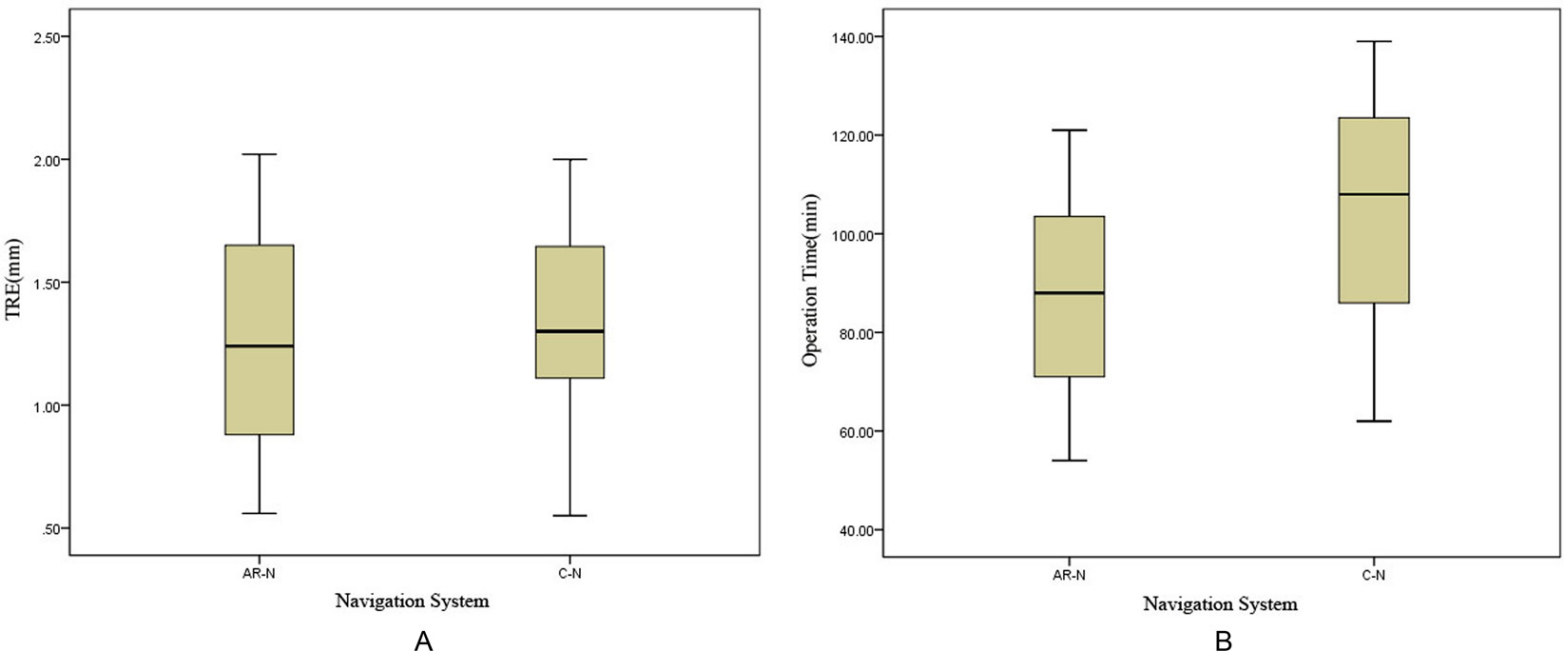
The target registration error (TRE) and the operation time (OT) for the augmented reality navigation (AR-N) system and conventional navigation (C-N) system. The performances of the AR-N and C-N systems during the simulated operations were compared. (A) There was no significant difference between the systems in TRE (*P* > 0.05), (B) but a significant difference was found in OT (*P* < 0.05).

The cadaver head experiment further demonstrated the effectiveness of the display effect of the AR-N system in an anatomical, structural environment. Fig 7 shows the seamless fusion of semitransparent endoscopic images with 3-D virtual images with satisfying anatomical structure contour continuity at the boundary. The two types of images changed synchronously when the endoscope moved and rotated in any direction. By adjusting the transparency of the endoscopic image or the virtual image rendering method, the operator is able to clearly observe the anatomical structures (e.g., the arteries) located in deep positions and around the surgical field (S2 Video). Table 2 compares the performance of the AR-N to the C-N system during the simulated operations; the average TRE rates were 1.28 ± 0.45 and 1.32 ± 0.41 mm, respectively, and the average OT were 88.27 ± 20.45 and 104.93 ± 24.61 min, respectively. Although we did not observe significant differences in TRE between the two systems (*P* > 0.05), task completion time differed significantly (*P* < 0.05) (Fig 8).

**Table 2:** Performance of augmented reality navigation (AR-N) system and conventional navigation (C-N) system during the simulated operations.

	Years of Experience	TRE (mm)		OT (min)	
		AR-N	C-N	AR-N	C-N
Participant 1	22	1.19	1.14	54	62
Participant 2	6	1.81	1.51	98	117
Participant 3	8	1.70	1.78	82	108
Participant 4	19	1.47	1.64	67	76
Participant 5	9	1.16	0.76	88	107
Participant 6	8	0.90	1.09	94	112
Participant 7	11	1.24	1.71	69	83
Participant 8	16	0.71	0.80	63	70
Participant 9	5	0.56	2.00	110	132
Participant 10	10	1.60	1.53	82	96
Participant 11	13	2.02	1.26	73	89
Participant 12	4	0.83	1.13	105	125
Participant 13	4	1.36	1.65	121	136
Participant 14	6	0.86	0.55	102	122
Participant 15	4	1.77	1.30	116	139
Average		1.28±0.45	1.32±0.41	88.27±20.45	104.93±24.61
		*P*>0.05		*P*<0.05	

**Fig 9:**
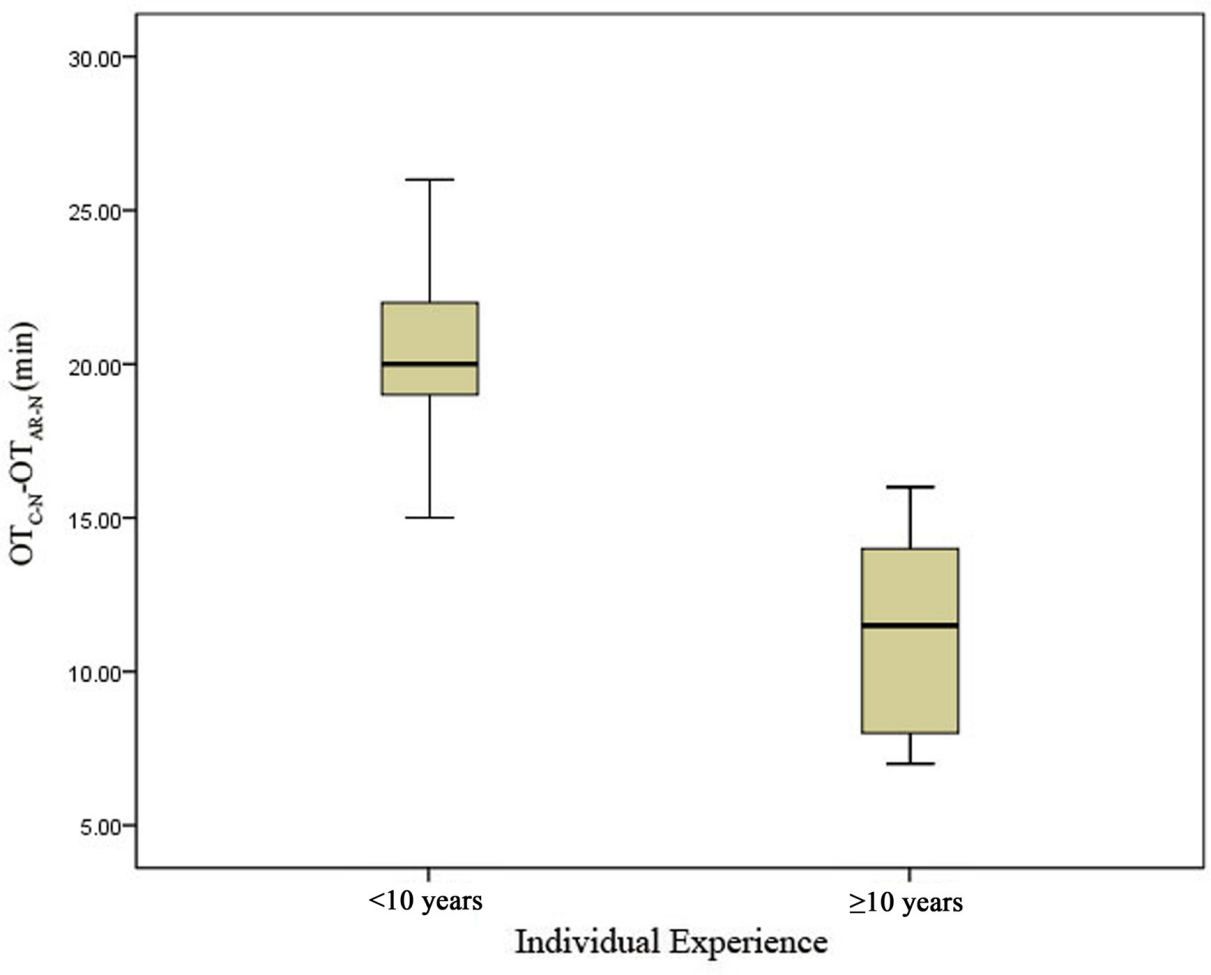
Reduction in operation time (OT) for the senior group compared with the junior group. The average difference in the time required for surgery (OT_C-N_-OT_AR-N_) was compared, and there was a significant difference between the two groups (*P* < 0.05).

**Fig 10:**
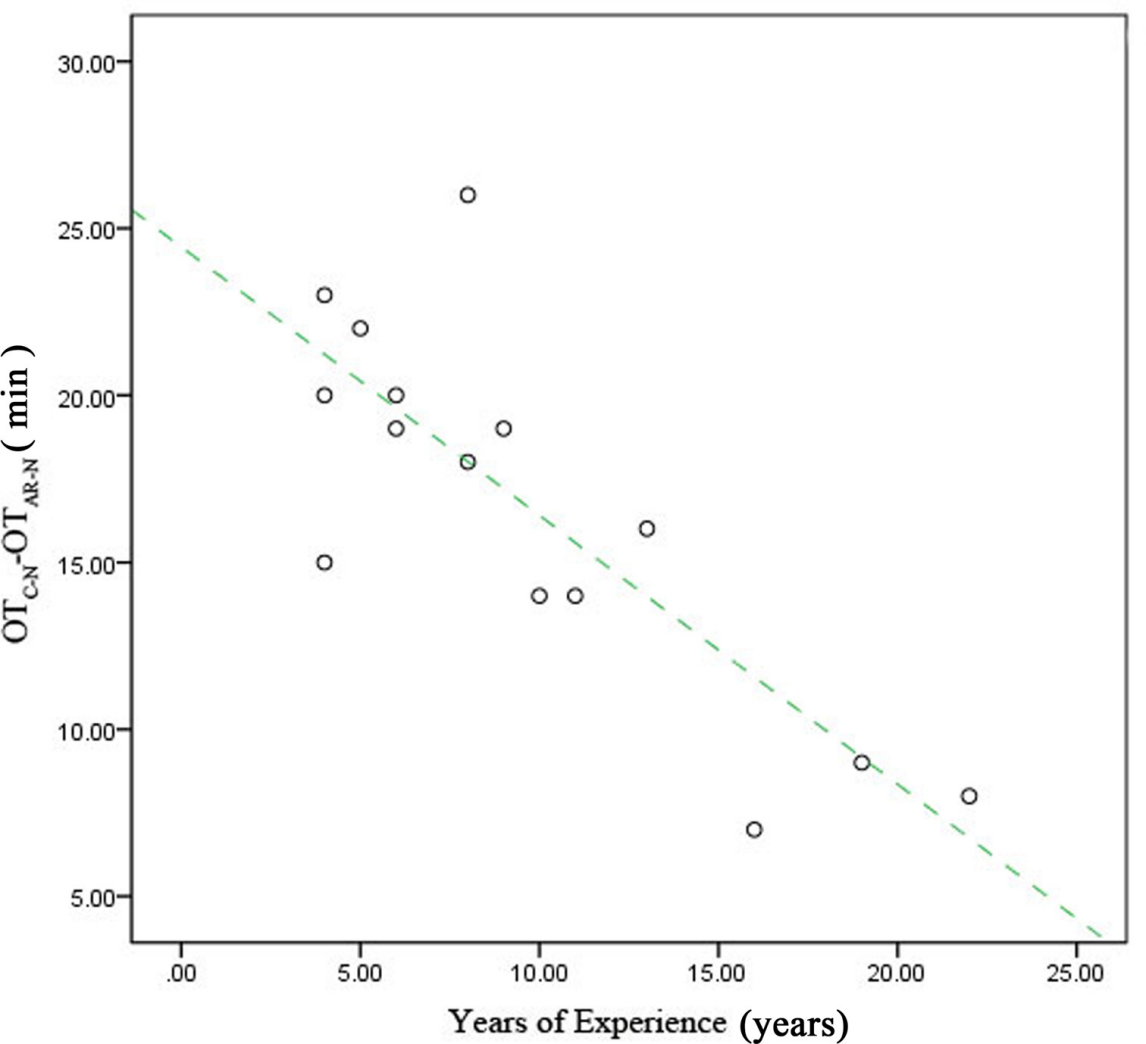
Reduction in operation time (OT) due to use of the augmented reality navigation system according to years of surgical experience. When we plotted the relationship between OT_C-N_-OT_AR-N_ (on the vertical axis) and individual experience (on the horizontal axis), a trend toward a negative correlation was found.

Performance was also analyzed according to surgeons’ years of experience. We divided the participants into two groups: a junior group (surgeons with <10 years of experience [6, 8, 9, 8, 5, 4, 4, 6, and 4 years; *n* = 9]) and a senior group (surgeons with ≥10 years of experience [22, 19, 11, 16, 10, and 13 y; *n* = 6]). The difference in time required for surgery between the two systems (OT_C-N_-OT_AR-N_) was greater for the junior group than for the senior group (*P* < 0.05) (Fig 9). Furthermore, the extent of time reduction decreased with increasing experience (Fig 10).

**Fig 11:**
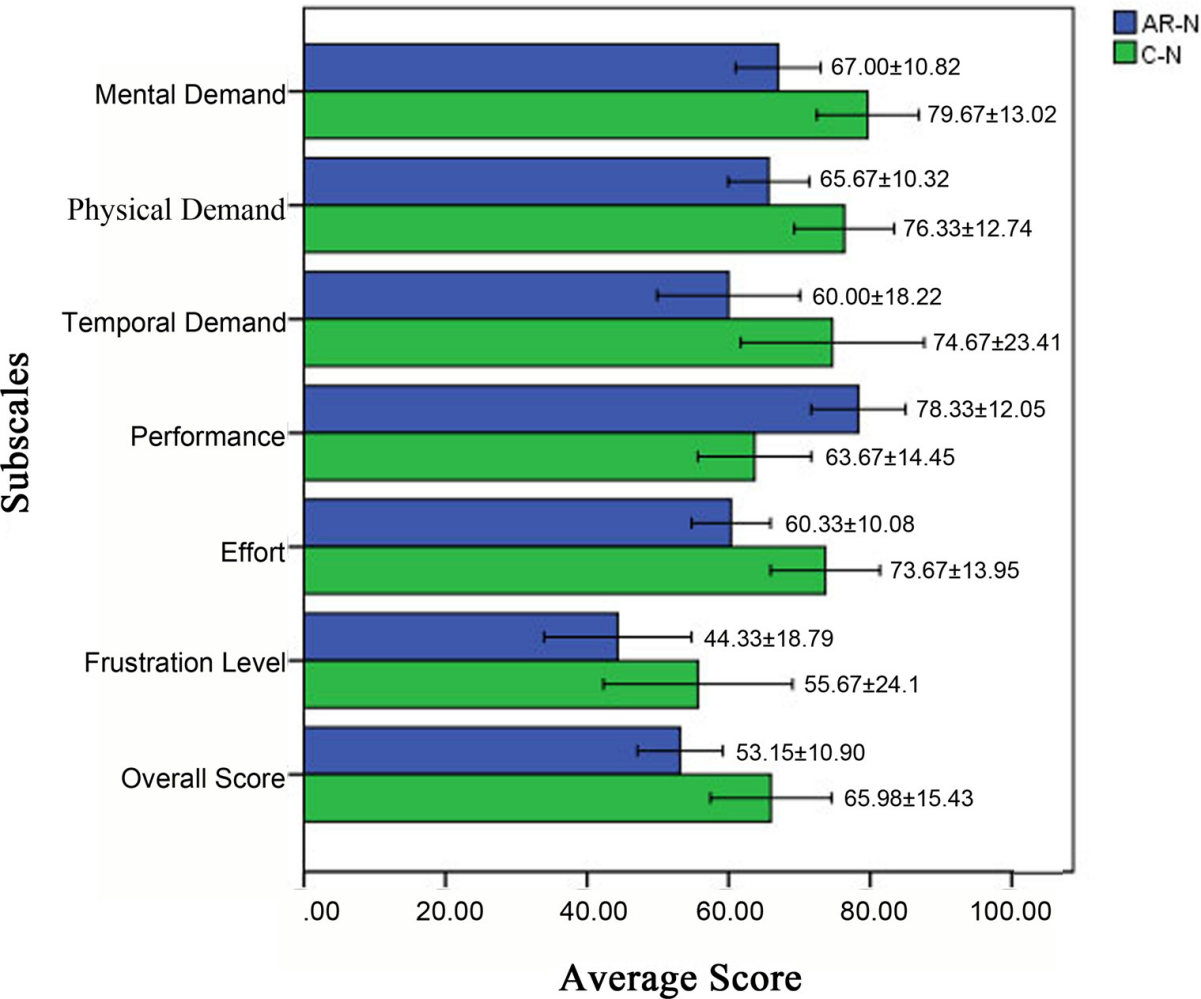
Differences in mental demand, physical demand, temporal demand, performance, effort, frustration level, and overall score between the two navigational systems. The augmented reality navigation (AR-N) system outperformed the conventional navigation (C-N) system on all items (*P* < 0.05).

All participants completed the computer-based survey as required after the simulated operation. The results showed that the average score on each item (mental demand, physical demand, temporal demand, performance, effort, frustration level, and overall scores) differed significantly (Wilcoxon rank sum test) between the AR-N and C-N systems (S2 Table), with the AR-N system outperforming the C-N system in all categories measured (Fig 11).

**Fig 12:**
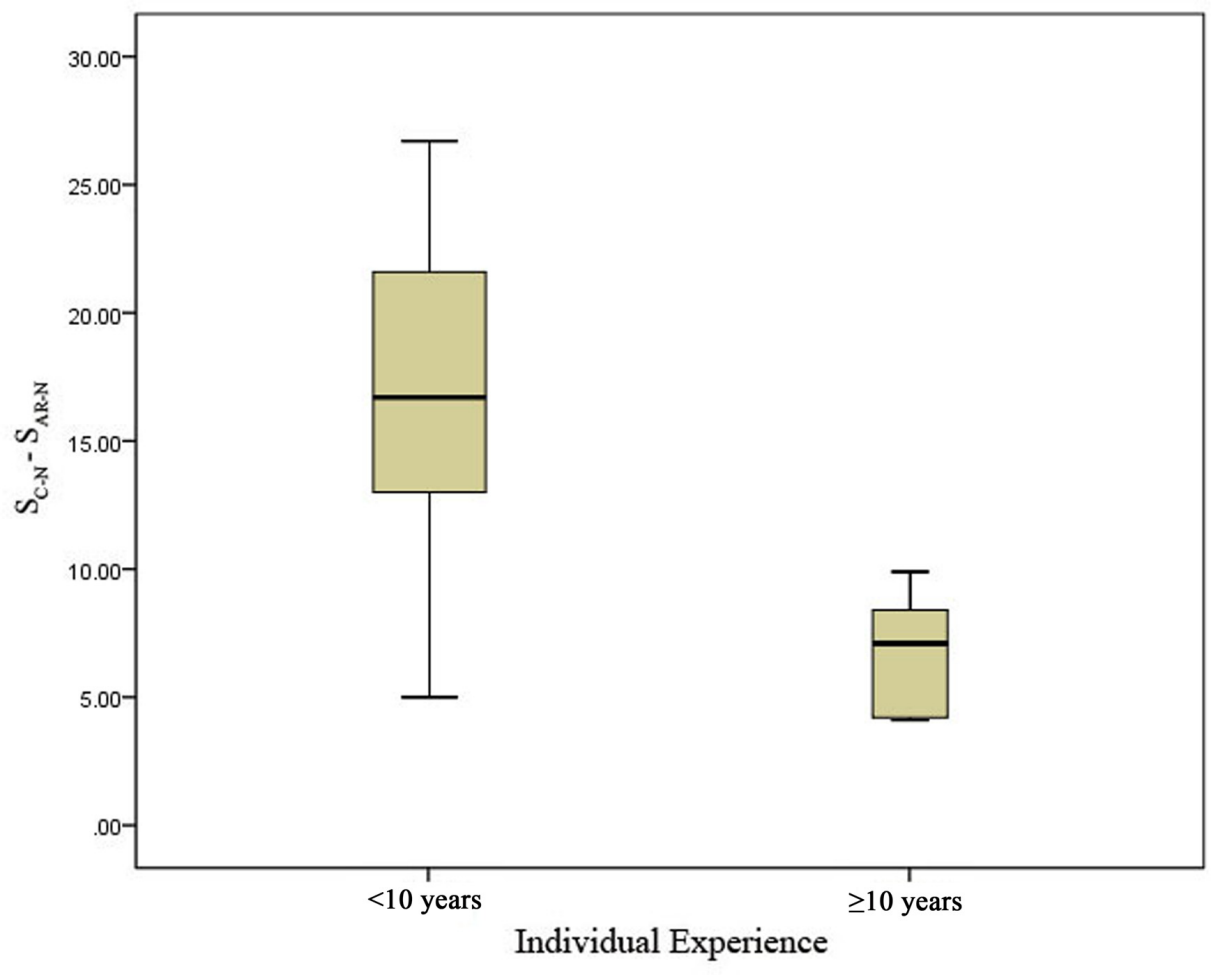
Reduction in mental workload in the senior group compared with the junior group. The average differences in the overall scores for mental workload during surgeries (S_C-N_-S_AR-N_) were compared, and there was a significant difference between the two groups (*P* < 0.05).

**Fig 13:**
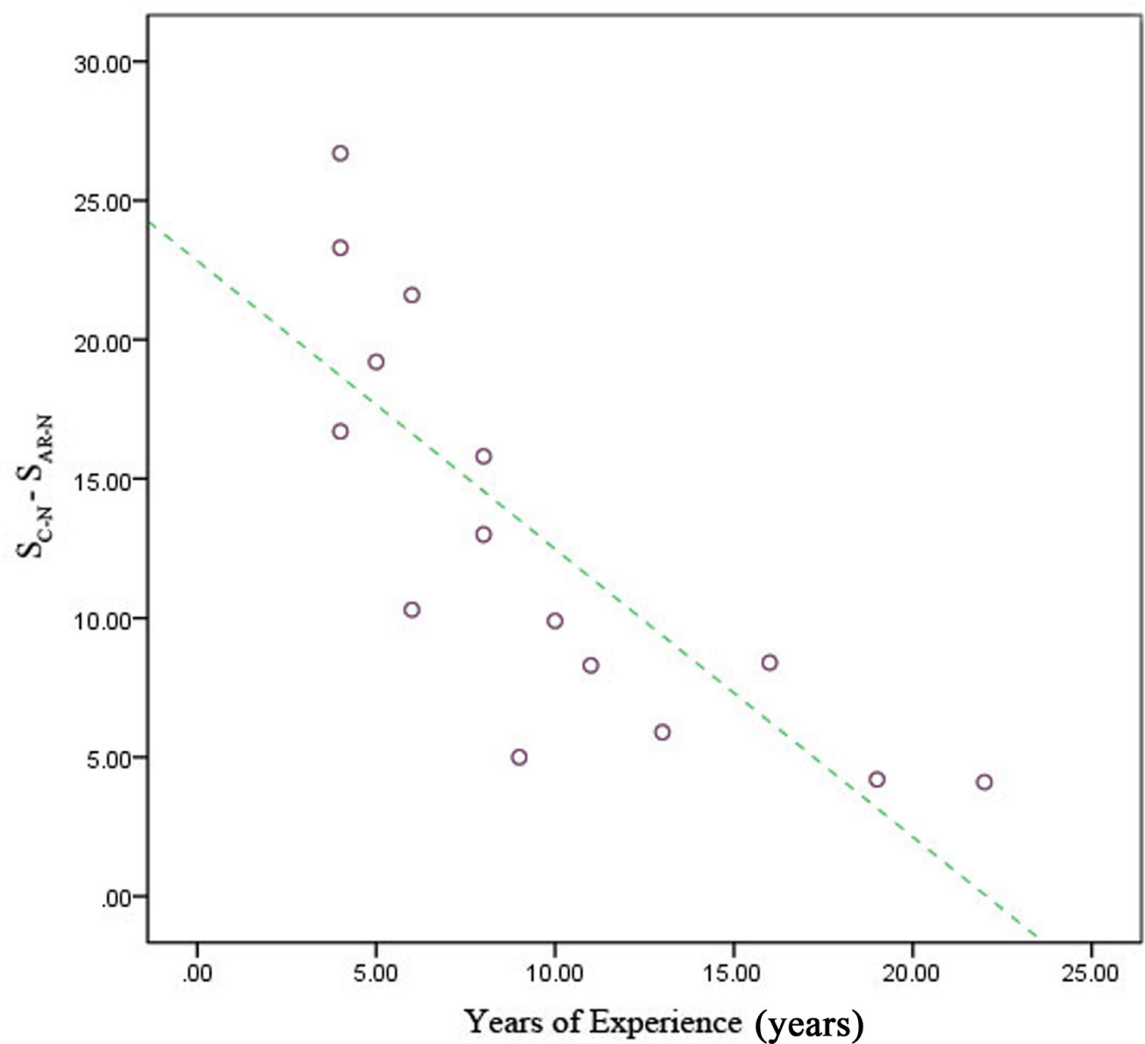
Reduction in surgeons’ mental workload when using the augmented reality navigation (AR-N) system according to years of surgical experience. When we plotted the relationship between S_C-N_-S_AR-N_ (on the vertical axis) and individual experience (on the horizontal axis), a trend toward a negative correlation was found.

When surgeons were divided by years of experience, the junior group had the greatest reduction in overall score for mental workload (S_C-N_-S_AR-N_) when using the developed navigational system compared with the senior group (*P* < 0.05) (Fig 12). This result was similar to that for operation time: the reduction in mental workload decreased with increasing experience (Fig 13).

## Discussion

Commensurate with a development period of more than 20 years, the performance of navigation systems during nasal endoscopic surgery has improved significantly. However, several recent studies have indicated that these systems may not be as effective as their users had expected. An evidence-based review by Ramakrishnan et al. [2] showed that the use of navigation systems was not directly associated with decreased surgical complications or improved surgical outcomes. One possible reason, according to our analysis, is that it is not easy for a doctor to match the tomographic image quickly and accurately to the endoscopic image in triplanar view mode of C-N system. Therefore, we considered the possibility that a navigation system based on the AR technique, which can reconstruct the stereoscopic form of surgical site and fuse the endoscopic image to 3-D reconstruction image, could be better able to meet the doctors’ observation habit. The most common application of AR techniques is in the field of neurosurgery, which requires a high degree of navigational accuracy [8]. More recently, AR techniques have been used during spinal [9], plastic [10], maxillofacial [11], and several other highly technical surgeries. However, few studies have focused on the application of AR to nasal endoscopic navigation. Although Caversaccio et al. [12], Thoranaghatte et al. [13], and Dixon et al. [14] reported dramatic improvements in this respect, the display mode of the navigation systems still fails to restore the original forms and positions of important structures within and surrounding the surgical field.

The navigational system for nasal endoscopic surgeries proposed in this study was developed based on the AR technique. It innovatively matches and semi-transparently fuses distortion-corrected nasal endoscopic images into 3-D virtual images based on preoperative imaging data. Thus, this system provides surgeons with a real-time stereoscopic image in a real sense, allowing surgeons to view deep anatomical structures, such as tumors, blood vessels, the brain, and eyeballs, in their original forms and positions by a superficial surgical field prior to sectioning. By operating in this manner, this system expands in depth and breadth the previously limited visual field of the nasal endoscope. Because some participating surgeons suggested that virtual images of bony structures in the display interface might interfere with observation of deeper structures, we made improvements to allow separate adjustment of the transparencies of real images, virtual images, and even different structures in virtual images.

In this study, we conducted head phantom and cadaver head experiments. The TRE was calculated during testing. Maurer et al. [15] and Citardi and Batra [16] suggest that this index could be the best indicator of the accuracy of a system. The results of the phantom experiment revealed that the theoretical accuracy of this system meets the recognized standard, while the cadaver head experiment revealed that the system was satisfactory in terms of display and accuracy in real operations. Furthermore, comparisons of the AR-N and C-N systems revealed that the AR-N system allowed surgeons to complete the simulated operation in less time. One possible explanation for this result is the improved display, which facilitates the cognitive processes required to connect imaging data to real structures and eliminates the need to look away from the screen or use probes to verify surgical sites.

Participants rated their mental workload, a key factor that affects a surgeon’s performance [7], during the operation using the NASA-TLX scale. The results indicate that using the AR-N system reduced mental workload compared to the C-N system. One possible explanation for this result is that this system allows participants to intuitively learn the anatomical structures within the surgical field, thus relieving mental stress during operation, reducing frustration, and increasing confidence.

Grouped analyses of experimental results indicated that doctors with less experience benefited more significantly from use of the AR-N system than surgeons with more experience, in terms of task completion time and mental workload. We suspect that this difference might be due to the anatomical knowledge already possessed by surgeons with more experience (i.e., surgeons who can easily connect imaging data to real images even under C-N monitoring). Although the intuitive process is not as well developed in less experienced doctors, a more intuitive display could improve this process.

In summary, the new AR-N system was accurate and intuitive for the proposed applications. It enables doctors to more easily learn the shape and positional relationships of anatomical structures in the surgical field, providing the necessary tools to achieve a more successful operation. Given these advantages, this system will likely play an important role in nasal endoscopic surgery, and specifically in endoscopic skull base surgery.

Besides, the system itself and the study has several limitations: (1) soft tissues might be displaced due to gravity or tumor resection during surgery, and navigational systems based on preoperative imaging data may not reflect tissue deformations. However, intraoperative CT/MRI devices to update imaging data may solve this problem; (2) in cadaver head experiment, we did not break down the task analysis by surgical sub-procedure, because we considered that in the initial validation, the overall differences in performance between the two systems may well depend on the differences in some key procedures. Thus we analyzed the task as a whole. In the next step of the research, we plan to compare the system performances in sub-procedures respectively, so as to find out the especial advantages of the AR-N system in complex surgical procedures.

Additionally, this study had a limited sample size. Further preclinical tests will need to be performed, and further improvements will be necessary according to the usage and habits of surgeons. We plan to combine this navigation system with a simulated surgical system based on force feedback technology. These integrated systems will be more appropriate for delivering the training necessary for skull-based surgery through an endoscopic endonasal approach, as well as for planning complex surgeries.

## Conclusion

The newly developed AR-N system uses a novel display mode that fuses corrected endoscopic images into a 3-D reconstructed background. Our findings indicate that this display mode can more successfully restore the original forms and positions of important structures within and surrounding the surgical field, and may thus provide surgeons with intuitive, real-time imaging information, which could ultimately reduce their judgment time and mental workload. This system might eventually help doctors perform safer operations based on more rational decisions. In addition, the AR-N system could be of interest to junior doctors being trained in endoscopic techniques: the anatomical information provided by the endonasal endoscope, the greater maneuverability of the instruments, and the clearly defined surgical steps may improve their learning efficiency. However, it should be noted that the navigation system serves as an adjunct to a surgeon’s skills and anatomical knowledge, not as a substitute [17]. Moreover, in cases of nasal and sinus lesions involving the brain or orbit, the assistance that can be provided by navigation systems is limited, such that multidisciplinary collaboration during the operation remains the preferred approach.

## Supporting Information

S1 Table The TRE (in mm) of the AR-N system during the head phantom experiment. (DOCX)

S2 Table Participants' mental workload scores following a simulated operation using two different navigational systems. (DOCX)

S1 Video Display fusing real endoscopic images to 3-D virtual images in the head phantom experiment. When we examined the left nasal cavity with the 0° lens, the contours of the anatomical structure (nasal bone, nasal septum, nasal turbinate, etc.) on both images overlapped accurately. We prefixed a small metallic ball to the clivus of the skull base to serve as a landmark, with the display indicating that the locations of the real and virtual ball were consistent with each other. Although the navigation image was partially delayed due to an issue with hardware configuration, the system could still be valuable with respect to the stereotaxy of anatomic structures and could be improved by upgrading the hardware in future work.
(MP4)

S2 Video Display of the AR-N system during the cadaver head experiment. The upper left section of the screen indicates the positional relationship between the lens and the head of the “patient”; the lower left section is the original endoscopic image, and the right section is the AR navigation image. The relative position of each section can be altered according to the operator’s requirements. Within the surgical field, the dura mater of the superior, posterior, and lateral walls of the sphenoid sinus were opened, and the anterior cerebral artery and left internal carotid artery were exposed; the actual locations were consistent with their projections, which were segmented from CT data. Virtual and real images were fused accurately and moved synchronously. The display expands the surgical field of nasal endoscopy in both depth and breadth and can thus provide operators with more intuitive information about anatomical structure, and forewarn them regarding possible future risks.
(MP4)
