# Supplementary material for: A Novel Augmented Reality Navigation System for Endoscopic Sinus and Skull Base Surgery: A Feasibility Study
Source: PLoS One. 2016 Jan 12;11(1):e0146996. doi: 10.1371/journal.pone.0146996 (PMC4710572; doi:10.1371/journal.pone.0146996)
Supplement: S1 Table — (DOCX) [file pone.0146996.s001.docx]

**S1 Table. TRE (in mm) of the AR-N system during the head phantom experiment.**

|  | 1 | 2 | 3 | 4 | 5 | 6 | 7 | 8 | 9 |
| --- | --- | --- | --- | --- | --- | --- | --- | --- | --- |
| Participant 1 | 1.16 | 1.14 | 1.57 | 0.84 | 1.41 | 1.21 | 1.10 | 1.08 | 0.54 |
| Participant 2 | 0.98 | 1.41 | 1.24 | 1.01 | 0.50 | 1.90 | 2.02 | 0.44 | 1.23 |
| Participant 3 | 0.73 | 0.86 | 1.87 | 1.23 | 1.76 | 1.22 | 2.10 | 1.33 | 1.34 |
| Participant 4 | 2.27 | 1.82 | 1.03 | 1.32 | 1.92 | 1.64 | 1.25 | 1.36 | 1.68 |
| Participant 5 | 1.03 | 0.90 | 1.05 | 1.39 | 0.65 | 0.92 | 0.51 | 1.23 | 1.12 |
| Participant 6 | 1.24 | 1.02 | 1.22 | 1.60 | 1.41 | 1.51 | 0.78 | 1.20 | 0.73 |
| Participant 7 | 1.45 | 0.50 | 1.51 | 0.83 | 0.69 | 1.44 | 1.43 | 1.07 | 0.60 |
| Participant 8 | 0.71 | 0.79 | 1.60 | 1.99 | 0.59 | 0.89 | 0.76 | 0.50 | 1.26 |
| Participant 9 | 1.16 | 1.06 | 1.63 | 1.10 | 1.36 | 0.78 | 1.39 | 1.32 | 1.34 |
