# Supplementary material for: A Novel Augmented Reality Navigation System for Endoscopic Sinus and Skull Base Surgery: A Feasibility Study
Source: PLoS One. 2016 Jan 12;11(1):e0146996. doi: 10.1371/journal.pone.0146996 (PMC4710572; doi:10.1371/journal.pone.0146996)
Supplement: S2 Table — (DOCX) [file pone.0146996.s002.docx]

**S2 Table. Participants' mental workload scores following a simulated operation using two different navigational systems.**

|  | Mental Demand | | Physical Demand | | Temporal Demand | | Performance | | Effort | | Frustration Level | | Overall Score | |
| --- | --- | --- | --- | --- | --- | --- | --- | --- | --- | --- | --- | --- | --- | --- |
|  | AR-N | C-N | AR-N | C-N | AR-N | C-N | AR-N | C-N | AR-N | C-N | AR-N | C-N | AR-N | C-N |
| Participant 1 | 45 | 55 | 55 | 65 | 35 | 30 | 85 | 75 | 55 | 60 | 30 | 25 | 39.2 | 43.3 |
| Participant 2 | 60 | 70 | 65 | 75 | 30 | 50 | 95 | 80 | 50 | 55 | 20 | 25 | 38.2 | 48.5 |
| Participant 3 | 75 | 90 | 70 | 85 | 75 | 95 | 60 | 50 | 70 | 90 | 80 | 80 | 68.3 | 81.3 |
| Participant 4 | 60 | 65 | 50 | 50 | 50 | 55 | 80 | 75 | 40 | 55 | 35 | 30 | 42.5 | 46.7 |
| Participant 5 | 80 | 85 | 50 | 55 | 45 | 50 | 90 | 85 | 45 | 50 | 20 | 25 | 41.7 | 46.7 |
| Participant 6 | 70 | 80 | 65 | 75 | 60 | 85 | 75 | 65 | 55 | 75 | 50 | 70 | 54.2 | 70.0 |
| Participant 7 | 55 | 65 | 60 | 75 | 40 | 50 | 80 | 75 | 65 | 70 | 20 | 25 | 43.3 | 51.6 |
| Participant 8 | 50 | 65 | 70 | 70 | 45 | 55 | 85 | 80 | 60 | 70 | 35 | 45 | 45.8 | 54.2 |
| Participant 9 | 70 | 85 | 60 | 85 | 75 | 90 | 75 | 55 | 55 | 75 | 60 | 80 | 57.5 | 76.7 |
| Participant 10 | 65 | 75 | 75 | 85 | 70 | 85 | 60 | 60 | 65 | 80 | 55 | 65 | 61.7 | 71.6 |
| Participant 11 | 75 | 85 | 80 | 80 | 90 | 95 | 55 | 45 | 75 | 80 | 60 | 65 | 70.8 | 76.7 |
| Participant 12 | 75 | 95 | 85 | 95 | 80 | 100 | 80 | 60 | 70 | 80 | 65 | 85 | 65.8 | 82.5 |
| Participant 13 | 80 | 95 | 60 | 75 | 75 | 95 | 80 | 35 | 70 | 100 | 45 | 80 | 58.3 | 85.0 |
| Participant 14 | 70 | 90 | 65 | 80 | 70 | 100 | 80 | 55 | 65 | 85 | 60 | 80 | 58.4 | 80.0 |
| Participant 15 | 75 | 95 | 75 | 95 | 60 | 85 | 95 | 60 | 65 | 80 | 30 | 55 | 51.6 | 74.9 |
| Average | 67.00±10.82 | 79.67±13.02 | 65.67±10.32 | 76.33±12.74 | 60.00±18.22 | 74.67±23.41 | 78.33±12.05 | 63.67±14.45 | 60.33±10.08 | 73.67±13.95 | 44.33±18.79 | 55.67±24.12 | 53.15±10.90 | 65.98±15.43 |
|  | *P*<0.05 | | *P*<0.05 | | *P*<0.05 | | *P*<0.05 | | *P*<0.05 | | *P*<0.05 | | *P<*0.05 | |

Note: Subscale scores in the table are quantified based on self-evaluation, which could be subjective and therefore should not be compared among individuals.
